# Effect of double-door laminoplasty on atypical symptoms associated with cervical spondylotic myelopathy/radiculopathy

**DOI:** 10.1186/s12893-016-0146-1

**Published:** 2016-05-10

**Authors:** Yuqing Sun, Aikeremujiang Muheremu, Kai Yan, Jie Yu, Shan Zheng, Wei Tian

**Affiliations:** Department of Spine Surgery, Beijing Ji Shui Tan Hospital, 31 Xinjiekou East Street, Xicheng District Beijing, 100035 China; Tsinghua University Medical Center, Room 330, Building5, Tsinghua University, Haidian District Beijing, 100035 China

**Keywords:** Cervical spondylotic myelopathy/radiculopathy, Atypical symptoms, Double-door laminoplasty

## Abstract

**Background:**

Double-door laminoplasty is an effective method in treating patients with cervical spondylosis. Many patients with cervical spondylosis experience a set of atypical symptoms such as vertigo and tinnitus, and wish to know if the surgical treatment for cervical spondylosis can also alleviate those symptoms. The current research was carried out to investigate if atypical symptoms can be alleviated in patients who received laminoplasty for the treatment of cervical spondylosis.

**Methods:**

One hundred ninety patients who received laminoplasty to treat cervical spondylotic myelopathy/radiculopathy in our center and complained about one or more of the atypical symptoms before the surgery were followed for a mean of 61.9 months (from 39 to 87 months) after the surgery. Severity scores were retrospectively collected by follow up outpatient visits or phone interviews. The data was calculated based on patient feedback on the frequency and severity of those symptoms before the surgery and at last follow up, and were compared by paired sample t-tests.

**Results:**

Most patients reported that the atypical symptoms such as vertigo (*P* <0.001), nausea (*P* <0.001), headache (*P* <0.001), tinnitus (*P* = 0.001), blur vision (*P* = 0.005), palpitation (*P* <0.001) and gastrointestinal discomfort (*P* = 0.001) were significantly alleviated at the last follow up; there was no significant change in the severity of hypomnesia (*P* = 0.675).

**Conclusion:**

Double-door laminoplasty can significantly alleviate most of the atypical symptoms in patients with cervical spondylosis. Further research is needed to explore mechanisms underlying this extra benefit of laminoplasty.

## Background

Cervical spondylosis is the most important cause of cervical spine related dysfunction among the elderly. In our clinical practice, besides typical symptoms such as neck and shoulder pain, numbness or hypersensitivity of arms and impairment of the fine-motor performance of extremities, patients with cervical spondylosis often complain about some atypical symptoms such as vertigo, headache, nausea, tinnitus, blurred vision, palpitation, hypomnesia and gastrointestinal discomfort. Since those symptoms were reported by a significant portion of the patients, but usually no typical radiologic or pathologic abnormalities that could lead to those symptoms, we call them “atypical” in the our study. Patients with atypical symptoms associated with cervical myelopathy/radiculopathy often wish to know if the surgical manipulation for cervical spondylosis can also alleviate the atypical symptoms. However, there is only a handful of rese/arch on this topic in the current literature [1].

Double-door laminoplasty was reported to be an effective method for treating patients with cervical spondylotic myelopathy [2]. It can achieve decompression on spinal cord and nerve roots while preserving the stability of the cervical spine [3, 4]. However, there is no literature on the efficacy of laminoplasty on the atypical symptoms associated with cervical spondylotic myelopathy/radiculopathy. We have found by long term follow up visits that patients with those symptoms may achieve improvements after laminoplasty. In the current study, we have analyzed the severity of these symptoms on patients with cervical spondylotic myelopathy/radiculopathy before they underwent laminoplasty and at the last follow up to evaluate the effectiveness of laminoplasty in alleviating the atypical symptoms.

## Methods

Among the patients diagnosed with cervical spondylotic myelopathy/radiculopathy, those who were suffering from the atypical symptoms and showed the indications for double-door laminoplasty were included in the study. The study was approved by the ethical committee of Beijing Ji Shui Tan Hospital. Patients provided written informed consent for the publication of their individual clinical details, and the procedures were in compliance with Helsinki Declaration.

### Inclusion criteria

(1) Reported typical symptoms of cervical spondylotic myelopathy/radiculopathy such as neck and shoulder pain, numbness or hypersensitivity of arms, impairment of the fine-motor performance of hands, difficulty in fast and coordinated movement of lower extremities, and abnormal reflex of extremities. Those symptoms were not alleviated in spite of systematic conservative treatment for more than a month. (2) Three or more levels of cervical spinal cord compression were shown by computed tomography and magnetic resonance imaging studies; (3) Aside from manifestations of typical cervical myelopathy, patient also presented with some atypical symptoms such as vertigo, tinnitus, headache, palpitation, blurred vision, hypomnesia, nausea and vomiting; (4) No abnormalities were found after consulting specialists from neurology, ophthalmology, cardiovascular otolaryngology; (5) Patient had no history of cervical surgery. (6): Patient agreed to receive double-door laminoplasty and cooperate with follow up visits after the surgery.

### Data collection

Data was collected during the years 2012 and 2014 by follow up outpatient visits or by phone interviews. All patients finished a questionnaire describing the atypical symptom severity before the surgery and at the last follow up, In the questionnaire, patients were asked to rate the frequency and severity of the atypical symptoms they were suffering (Table 1).

**Table 1:** Score sheet used in the current research to describe the frequency and severity of atypical symptoms

Frequency	0	never have the symptom
	1	have the symptom occasionally
	2	have the symptom often
	3	have the symptom all the time
Severity	0	does not exist
	1	mild
	2	moderate
	3	severe

### Statistics

For statistical analysis, the overall severity of atypical symptoms in patients with cervical spondylotic myelopathy/radiculopathy were calculated by the frequency and severity provided by the patients using the evaluation criteria in (Table 2).

**Table 2:** Score sheet used in the current research to calculate overall severity score according to the severity and frequency scores provided by the patients

Overall severity score	Corresponding severity and frequency scores provided by the patients
0	frequency score: 0 and severity score: 0
1	frequency score:1 and severity score:1
2	frequency score:1 and severity score:2; or frequency score:2 and severity score:1
3	frequency score:2 and severity score:2; or frequency score:3 and severity score:1
4	frequency score:3 and severity score:2; or frequency score:1 and severity score:3
5	frequency score:2 and severity score:3; or frequency score:3 and severity score:3

Overall severity scores of the atypical symptoms before and after surgery were compared by paired-sample t-tests using SPSS17.0 statistical software (Chicago Illinois, USA).

## Results

### General results

One hundred ninety patients treated from the Feb 2002 to Nov 2011 reported atypical symptoms including one or more of vertigo, tinnitus, headache, palpitation, blurred vision, hypomnesia, nausea besides typical symptoms of cervical myelopathy (Table 3). There were 143 males and 47 females with the age of 54.5 ± 8.6 (range from 26 to 76). 157 patients were diagnosed with myelopathy, 12 with radiculopathy and 21 with radiculomyelopathy. The mean follow-up was 61.9 months (range from 39 to 87 months).

**Table 3:** Demographic characteristics of the patients included in the study

	Vertigo	Headache	Nausea	Tinnitus	Blur vision	Palpitation	Hypomnesia	Gastointestinal	Type 1/2/3	M/F
Patient Number	75	65	27	72	46	87	42	47	157/12/21	143/47
Percentage	39.5	34.2	14.2	37.9	24.2	45.8	22.1	24.7	83/6/11	75/25

Type 1: cervical myelopathy, Type 2: cervical radiculopathy, Type 3: Cervical Radiculomyelopathy

### Severity of atypical symptoms

At the last follow up, Most of the patients achieved full or partial recovery after the surgery (Table 4).

**Table 4:** Patient numbers with different treatment results

	Vertigo	Headache	Nausea	Tinnitus	Blur vision	Palpitation	Hypomnesia	Gastroinestinal	Total
Cases	75	65	27	72	46	87	41	47	190
Healed	47	34	19	24	13	27	3	19	35
Better	10	14	2	6	5	11	1	4	96
Same	14	16	5	36	25	41	35	22	42
Worse	4 (2 new)	1	1	6 (5 new)	3	8 (1new)	2 (1new)	2 (1new)	17

According to the frequency and severity of each atypical symptoms reported by the patients, overall severity scores of the patients were calculated. According to the paired sample t- tests, significant alleviation was achieved with vertigo (*P* <0.001), headache (*P* <0.001), nausea (*P* <0.001), tinnitus (*P* = 0.001), blur vision (*P* = 0.005), palpitation (*P* <0.001) and gastrointestinal discomfort (*P* = 0.001). There was no significant change with hypomnesia (*P* = 0.675). The total overall severity score of sympathetic symptoms at the last follow up (2.96 ± 2.76) was significantly (*P* <0.001) lower than before the surgery (4.91 ± 3.61) (Table 5), confirming the effectiveness of double-door laminoplasty in alleviating the atypical symptoms in patients with cervical spondylotic myelopathy/radiculopathy.

**Table 5:** Results of paired sample t-tests comparing symptom severity scores before the surgery and at the last follow up

	Vertigo	Headache	Nausea	Tinnitus	Blur vision	Palpitation	Hypomnesia	Gasterointestinal	Total
SBS	2.00 ± 1.17	2.02 ± 1.11	1.89 ± 1.37	2.44 ± 1.44	1.93 ± 1.02	1.69 ± 0.96	2.76 ± 1.22	1.89 ± 1.31	4.91 ± 3.61
SAL	0.68 ± 1.10	0.71 ± 0.89	0.46 ± 0.92	1.77 ± 1.68	1.43 ± 1.31	1.24 ± 1.15	2.71 ± 1.27	1.04 ± 1.32	2.96 ± 2.76
SAL- SAS	1.32 ± 1.59	1.31 ± 1.30	1.43 ± 1.50	0.67 ± 1.64	0.50 ± 1.15	0.45 ± 1.10	0.05 ± 0.74	0.85 ± 1.64	1.95 ± 2.96
t	7.20	7.91	5.04	3.49	2.95	3.76	0.42	3.55	9.09
P	<0.001	<0.001	<0.001	0.001	0.005	<0.001	0.675	0.001	<0.001

Scores were demonstrated as: Mean ± Standard deviation. Abbreviations: *SBS* score before surgery, *SAL* score at the last follow up

## Discussion

Cervical spondylosis is the most prominent cause of spinal cord related dysfunction among people over 55 years of age [5, 6]. Degenerative changes of cervical intervertebral disk, lamina, foramen, ossification of posterior longitudinal ligament and ligamentum flavum could compress to the spinal cord and nerve roots, which impairs sensory and motor functions of trunk and extremities. Early stage clinical symptoms of cervical spondylosis include neck and shoulder pain, packing feeling of the trunk, numbness or hypersensitivity of arms, impairment of the fine-motor performance, difficulty in fast and coordinated movement of lower extremities, abnormal reflex of extremities. Patients with later stage cervical spondylosis may also suffer from difficulty in steady walking, active reflex of tendons as well as atrophy of related muscles [7–10].

In our clinical practice, patients with cervical spondylosis often complain about a set of symptoms including vertigo, tinnitus, palpitation, headache, blurred vision, hypomnesia and nausea. It was first reported by Barré and liéou in 1926, and this set of symptoms was also called “Barré and liéou syndrome” [11]. We note that although a considerable number of patients experience those symptoms, except from the degenerative changes in the cervical spinal region, no radiologic and pathologic studies could find any typical abnormalities that can be the cause of those symptoms, so we call them “atypical symptoms” in this study [12].

There is a great amount of literature about cervical spondylosis, however, few studies were carried out regarding the mechanisms or treatment strategies for atypical symptoms so far. In patients with such symptoms, cervical disc degeneration and ligament ossification are the only abnormalities that can be observed by iconographic examinations. Some authors also assumed that those symptoms may be the result of mechanical compression surrounding tissues on vertebral artery [13] and the sympathetic nerves [14–16] in the artery. Instability of the cervical spine and whiplash injury [17, 18] has also been reported to be associated with those symptoms. However, there is no consensus on the molecular, cellular or pathologic mechanisms of the atypical symptoms.

To our knowledge, the only hypothesis with solid clinical and pathological evidence is that the atypical symptoms could be caused by the stimulation of sympathetic nerves in the posterior longitudinal ligament [19, 20]. The fact that a network of sympathetic nerves were found in the posterior longitudinal ligament in several cadaveric and animal studies [21–24], and that relieving the compression of herniated intervertebral disks on the posterior longitudinal ligament or removing part of the posterior longitudinal ligament have effectively alleviated headache in patients who received anterior cervical discectomy and fusion (ACDF) or total disk replacement (TDR) surgery [12, 25–27] may be the proof for this hypothesis. However, according to the results of the current study and a previous study from our group [28], atypical symptoms were completely or partially relieved after the patients underwent double-door laminoplasty. Considering that no herniated intervertebral disks were ablated, and that the posterior longitudinal ligament was intact during the laminoplasty, we hypothize that stimulation of sympathetic nerves in the posterior longitudinal ligament may not be the only reason for the appearance of atypical symptoms in patients with cervical spondlotic myelopathy/radiculopathy. Considering that one thing in common among all ACDF, TDR and laminoplasty surgeries is the dicompression of spinal cord, we assume that atypical symptoms in patients with cervical spondylotic myelopathy/radiculopathy might be the results of compression on the spinal cord itself. Further research should be carried out to explore the underlying mechanism of this phenomenon.

## Conclusion

It can be concluded from the current study that bilateral double-door laminoplasty can relieve some atypical symptoms such as vertigo, headache, nausea, tinnitus, blurred vision and palpitation in patients with cervical spondylotic myelopathy/radiculopathy.

## Declarations

### Ethics approval and consent to participate

The study was approved by the ethical committee of Beijing Ji Shui Tan Hospital. Patients provided written informed consent for the publication of their individual clinical details, and the procedures were in compliance with Helsinki Declaration.

### Availability of data and materials

The datasets supporting the conclusions of this article is are included within the article.

## Abbreviations

SBS: score before surgery
SAL: score at the last follow up
ACDF: anterior cervical discectomy and fusion
TDR: total disk replacement
